# Antibiotic Resistance and Toxin Production of *Clostridium difficile* Isolates from the Hospitalized Patients in a Large Hospital in Florida

**DOI:** 10.3389/fmicb.2017.02584

**Published:** 2017-12-22

**Authors:** Zhong Peng, Anteneh Addisu, Sally Alrabaa, Xingmin Sun

**Affiliations:** ^1^Department of Molecular Medicine, Morsani College of Medicine, University of South Florida, Tampa, FL, United States; ^2^State Key Laboratory of Agricultural Microbiology, College of Veterinary Medicine, Huazhong Agricultural University, Wuhan, China; ^3^Department of Internal Medicine, Morsani College of Medicine, University of South Florida, Tampa, FL, United States

**Keywords:** *Clostridium difficile*, toxin-type, antibiotic resistance, toxin production, broth microdilution

## Abstract

*Clostridium difficile* is an important cause of nosocomial acquired antibiotic-associated diarrhea causing an estimated 453,000 cases with 29,000 deaths yearly in the U.S. Both antibiotic resistance and toxin expression of *C. difficile* correlate with the severity of *C. difficile* infection (CDI). In this report, a total of 139 *C. difficile* isolates from patients diagnosed with CDI in Tampa General Hospital (Florida) in 2016 were studied for antibiotic resistance profiles of 12 types of antibiotics and toxin production. Antibiotic resistance determined by broth microdilution method showed that strains resistant to multi-antibiotics are common. Six strains (4.32%) showed resistance to six types of antibiotics. Twenty strains (14.39%) showed resistance to five types of antibiotics. Seventeen strains (12.24%) showed resistance to four types of antibiotics. Thirty-nine strains (28.06%) showed resistance to three types of antibiotic. Thirty-four strains (24.46%) showed resistance to two types of antibiotics. While, all isolates were susceptible to metronidazole, and rifaximin, we found that one isolate (0.72%) displayed resistance to vancomycin (MIC ≥ 8 μg/ml), and another one was resistant to fidaxomicin (MIC >1 μg/ml). The percentage of isolates resistant to cefoxitin, ceftriaxone, chloramphenicol, ampicillin, clindamycin, erythromycin, gatifloxacin, and moxifloxacin was 75.54, 10.79, 5.76, 67.63, 82.70, 45.32, 28.06, and 28.78%, respectively. Toxin profiling by PCR showed the isolates include 101 (72.66%) A+B+CDT-strains, 23 (16.55%) A+B+CDT+ strains, 3 (2.16%) A-B+CDT+ strains, 1 (0.72%) A-B+CDT-strains, and 11 (7.91%) A-B-CDT-strains. Toxin production determined by ELISA using supernatants of bacterial culture harvested at 12, 24, 48, and 72 h of post inoculation (hpi) showed that the toxins were mainly produced between 48 and 72 hpi, and toxin B (TcdB) was produced faster than toxin A (TcdA) during the experimental time (72 hpi). In addition, the binary-positive strains were likely to yield more toxins compared to the binary-negative strains. This work contributes to the current understanding of the antibiotic resistance and virulence of *C. difficile* clinical strains.

## Introduction

*Clostridium difficile* infection (CDI) is responsible for over 500,000 enteric infections, and caused an annual economic burden ranging from $436 million to $3 billion dollars in the US (Napolitano and Edmiston, 2017). More worrisome, incidence, and severity are increasing, which is in part associated with the emergence and prevalence of a fluoroquinolone-resistant *C. difficile* clone known as restriction endonuclease type BI/pulsed-field type NAP1, toxinotype III, or polymerase chain reaction (PCR) ribotype 027 *C. difficile* (Lim et al., 2014; Napolitano and Edmiston, 2017).

Currently, CDI treatment mainly relies on three antibiotics including metronidazole, vancomycin, and fidaxomicin (Cohen et al., 2010; Leffler and Lamont, 2015). While effective, *C. difficile* isolates with significantly reduced susceptibility and even resistance to these antibiotics have been continuously reported (Peng et al., 2017). In addition, the use of many other antibiotics is thought to be the most important risk factor for CDI. Many antibiotics such as ampicillin, amoxicillin, cephalosporins, clindamycin, and fluoroquinolones have been proposed to be associated with the disease (Leffler and Lamont, 2015; Peng et al., 2017). In this regard, continuous monitoring of the antibiotic resistance in *C. difficile* isolates from patients will be essential in understanding epidemiology and evolution of *C. difficile*, especially in the aspect of antibiotics resistance.

The principle factor for the development of CDI symptoms is the production of two main toxins: toxin A (TcdA) and toxin B (TcdB) (Napolitano and Edmiston, 2017). Their encoding genes *tcdA* and *tcdB* were harbored within the known pathogenicity locus (PaLoc) in *C. difficile* genome (Dingle et al., 2014). In addition to those two large toxins, ~20% *C. difficile* strains including the epidemic 027 strain are found to express the third toxin, the binary toxin (CDT), which is encoded within a locus (CdtLoc) physically separated from the PaLoc (Eckert et al., 2015; Roy Chowdhury et al., 2016). Previous data showed that patients infected with strains producing CDT had ~60% higher fatality rates than those infected with CDT-deficient strains (Bacci, 2011), and that CDT was found to enhance *C. difficile* virulence by suppressing protective colonic eosinophilia (Cowardin et al., 2016). Therefore, profiling toxin production of *C. difficile* clinical isolates is also important in understanding the evolution of pathogenicity of *C. difficile*.

In this report, a total of 139 *C. difficile* strains isolated from the fecal samples of patients with CDI in Tampa General Hospital (TGH) in 2016 were screened for antibiotic resistance and toxin production.

## Materials and methods

### Bacterial strains and cultural conditions

A total of 139 *C. difficile* isolates from patients diagnosed with CDI in TGH (Florida, USA) in 2016 were used in this study. *C. difficile* strains were cultured in BHIS medium at 37°C under anaerobic condition. To determine the toxin production, the TY medium (3% w/v tryptose, 2% w/v yeast extract, 0.1% w/v thioglycollate, PH 7.4) was used, which was reported to increase toxin yield (Sorg and Dineen, 2009). For broth microdilution assays determining minimum inhibitory concentrations (MIC), brucella broth medium was used (CLSI, 2009).

### Profiling of *c. difficile* toxin genes by PCR

PCR assays were carried out using the bacterial genomic DNA or *C. difficile* culture supernatants (Hiraishi, 1992) as template, following the instructions of a Q5® High-Fidelity PCR Kit (New England BioLabs, USA). Toxin-encoding genes including *tcdA, tcdB, cdtA*, and *cdtB* were detected using a 5-plex PCR method established by Persson et al. (2008). Primers were listed in Table 1. The reaction was performed in a 25 μl mixture containing template DNA (5 μl), Q5 DNA High-Fidelity 2 × Master Mix (5 μl), primers with final concentrations listed in Table 1, and then added nuclease free water to 25 μl. Thermo cycles were 98°C, 30 s; 35 cycles for 98°C, 10 s; 54°C, 45 s; 72°C, 80 s; final extension at 72°C, 10 min. PCR products were analyzed by electrophoresis on a 3% agarose gel.

**Table 1:** Primers for detecting the 16S rDNA and toxin-encoding genes of Clostridium difficile^*^.

**Gene target**	**Sequence (5′-3′)**	**Concentration (μM)**	**Product size (bp)**
*tcdA*	GCATGATAAGGCAACTTCAGTGGTA	0.6	629
	AGTTCCTCCTGCTCCATCAAATG	0.6	
*tcdB*	CCAAARTGGAGTGTTACAAACAGGTG	0.4	410
	GCATTTCTCCATTCTCAGCAAAGTA	0.2	
	GCATTTCTCCGTTTTCAGCAAAGTA	0.2	
*cdtA*	GGGAAGCACTATATTAAAGCAGAAGC	0.05	221
	GGGAAACATTATATTAAAGCAGAAGC	0.05	
	CTGGGTTAGGATTATTTACTGGACCA	0.1	
*cdtB*	TTGACCCAAAGTTGATGTCTGATTG	0.1	262
	CGGATCTCTTGCTTCAGTCTTTATAG	0.1	
*16SrDNA*	GGAGGCAGCAGTGGGGAATA	0.05	1062
	TGACGGGCGGTGTGTACAAG	0.05	

* *Adopted from Persson et al. (2008)*.

### Determination of antibiotic resistance

MIC of 12 types of antibiotics including metronidazole (MTZ), vancomycin (VAN), rifaximin (RFX), fidaxomicin (FDX), cefoxitin (FOX), ceftriaxone (CRO), chloramphenicol (CHL), ampicillin (AMP), clindamycin (CLI), erythromycin (ERY), gatifloxacin (GAT), and moxifloxacine (MXF) were determined using broth microdilution assays according to Clinical and Laboratory Standards Institute (CLSI) guidelines (document M11-A7) (CLSI, 2009). A series of two-fold dilutions of each antibiotic with final concentrations ranging from 0 to 256 μg/ml was made in a 96-well plate in pre-reduced supplemented Brucella broth. Interpretation of testing results were based on CLSI M100-S25 (Patel et al., 2015), while the MIC results for vancomycin (resistance ≥ 8 μg/ml), rifaximin (resistance ≥ 32 μg/ml), fidaxomicin (intermediate resistance >1 μg/ml), erythromycin (resistance ≥ 8 μg/ml), and gatifloxacin (resistance ≥ 8 μg/ml) were interpreted resistance, respectively, as previously described (O'Connor et al., 2008; Spigaglia et al., 2008; Huhulescu et al., 2011; Freeman et al., 2015; Álvarez-Pérez et al., 2017). Parallel tests were performed for the confirmation of the final results. Interpretive criterions of the antibiotics used in this study were listed in Table 2.

**Table 2:** Interpretive criterions of the antibiotics used in this study.

	MTZ^*^	**VAN^§^**	**RFX^§^**	**FDX^§^**	FOX^*^	CRO^*^	CHL^*^	AMP^*^	CLI^*^	**ERY^§^**	GAT^*^	MXF^*^
S (μg/ml)	≤8	≤2	–	<1	≤16	≤16	≤8	≤0.5	≤2	–	≤2	≤2
I (μg/ml)	16	4	–	>1	32	32	16	1	4	–	4~7	4
R (μg/ml)	≥32	≥8	≥32	–	≥64	≥64	≥32	≥2	≥8	≥8	≥8	≥8

Breakpoints were defined as sensitive (S), intermediately resistant (I), or resistant (R) with reference to CLSI (^*^) or published data (^§^).

### Determination of toxin production

We measured toxin production at 12, 24, 48, 72 h post inoculation (hpi). Briefly, single colonies of the strains were initially cultured in BHIS medium and finally transformed into fresh TY medium at volume ratio of 1: 100 for inducing toxin expression (Sorg and Dineen, 2009). Strain cultures at each time point were re-suspended thoroughly prior to sampling. One milliliters of thoroughly re-suspended cultures from different strains at a given time point were removed, adjusted to the same OD600 value; and supernatants from different cultures after centrifugation at 12,000 rpm for 10 min were used for toxin determination. To determine toxin production by ELISA, 96-well plates were coated with 50 μl per well of anti-TcdA and anti-TcdB antibody at a concentration of 0.5 μg/ml. The coated plates were washed with PBST (washing buffer, 1 × PBS+0.05% Tween 20), and blocked with 150 μl per well of blocking buffer (PBS+5% dry milk) for 2 h. After being washed with PBST, the plates were incubated with 50 μl bacterial supernatants/well collected at 12, 24, 48, 72 h post inoculation in TY medium at room temperature for 1.5 h. After being washed with PBST, the plates were further incubated with HRP-Chicken anti-*C. difficile* Tcd A antibody (1: 5000 dilution, Gallus Immunotech, USA) or HRP-Chicken anti-*C. difficile* TcdB antibody (1: 5000 dilution, Gallus Immunotech, USA) per well at 37°C for 1 h. The plate was washed again, and each well was added 50 μl TMB substrate and incubated for 30 min at room temperature. Then reaction was finally stopped with 25 μl 2 N H_2_SO_4_, and OD_450_ was determined by a plate reader (BioTek *Synergy HT*, USA). Purified TcdA and TcdB were used as standards. Toxin concentrations at different time points were calculated according to standard curves generated from the toxin standards.

### Statistics analysis

Statistics analysis was performed using the “Two-way ANOVA” strategy in GraphPad Prism 6.0. Data represents mean ± SD. The significance level was set at *P* < 0.05.

## Results

### Toxin gene profiles of *c. difficile* isolates by PCR

**Figure 1:**
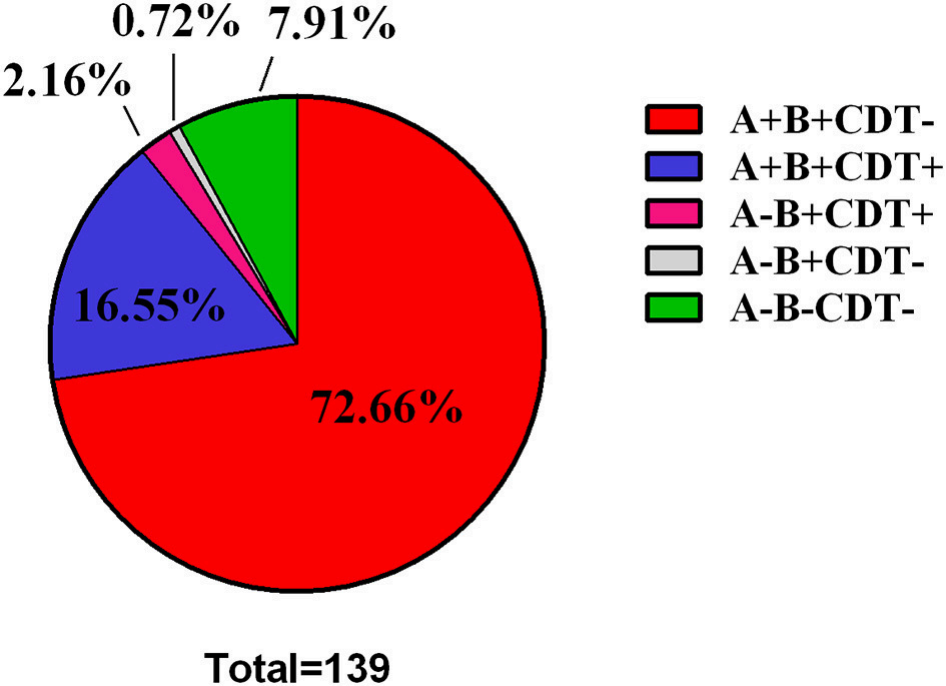
Pie Chart showing the distribution of toxin type among the 139 *C. difficile* clinical strains.

Of the 139 TGH clinical isolates, 128 strains (92.09%, *n* = 139) were determined to be toxigenic strains, while the rest 11 strains (7.91%, *n* = 139) were nontoxigenic strains (A-B-CDT-) (Figure 1, Table 3). Among the toxigenic strains, 101 strains (78.91%, *n* = 128) were positive for both *tcdA* and *tcdB* but negative for binary toxin encoding genes (A+B+CDT-); however, 23 strains (17.20%, *n* = 128) were positive for both *tcdA, tcdB*, and binary toxin encoding genes (A+B+CDT+). Particularly, three strains (2.34%, *n* = 128) were positive for *tcdB* and binary toxin encoding genes but negative for *tcdA* (A-B+CDT+). One strain (0.78%, *n* = 128) were found to be positive for *tcdB* but negative for both *tcdA* and binary toxin encoding genes (A-B+CDT-).

**Table 3:** Distribution of the toxin-encoding genes among the 139 *Clostridium difficile* clinical isolates.

**Toxin-type**	**A+B+CDT-**	**A+B+CDT+**	**A-B+CDT+**	**A-B+CDT-**	**A-B-CDT-**
No. of strains	101	23	3	1	11
Percentage (%)	72.66	16.55	2.16	0.72	7.91

### Toxin production of the *c. difficile* isolates

**Figure 2:**
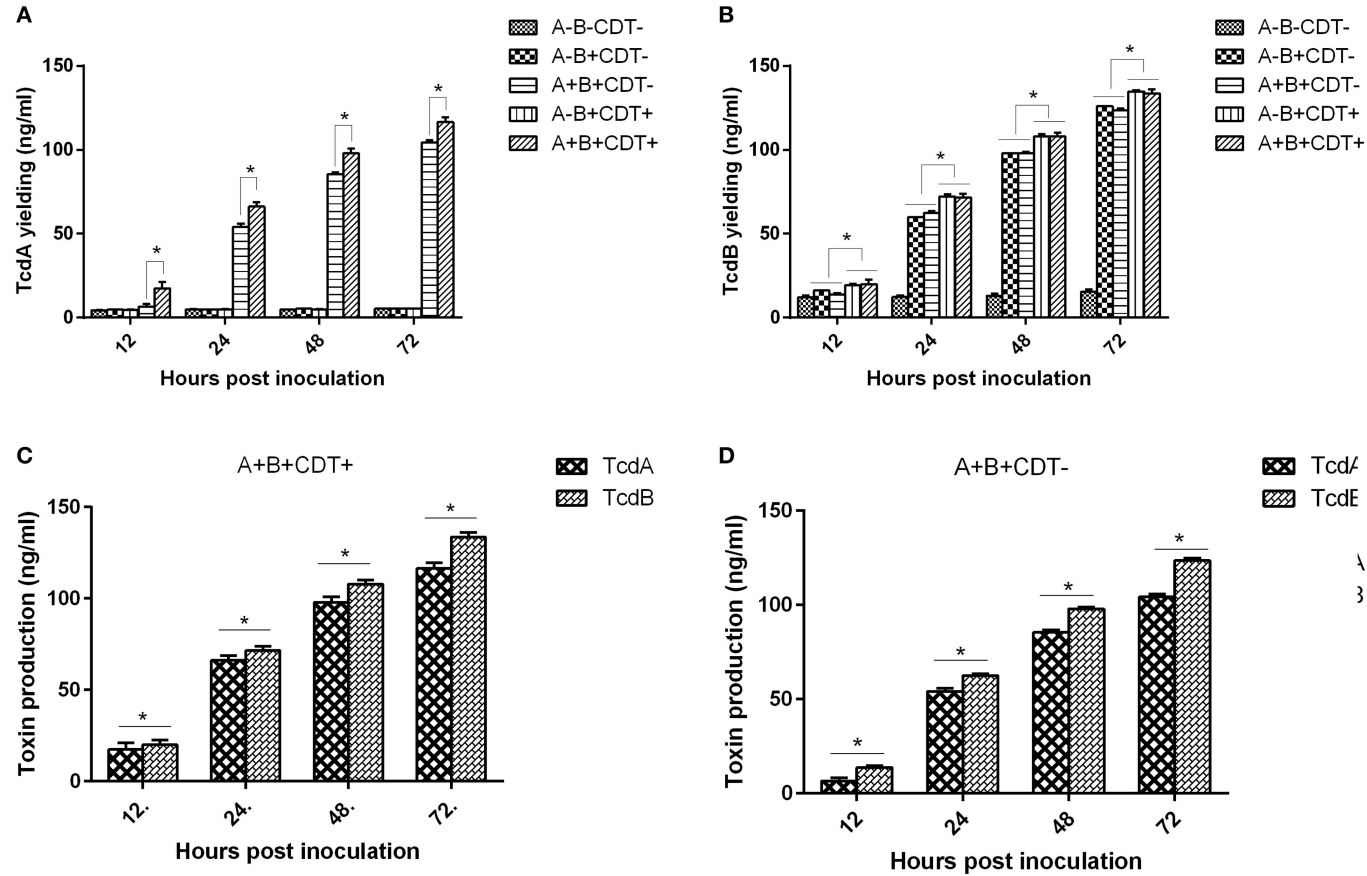
Average concentrations of TcdA and TcdB produced by *C. difficile* strains of different toxigenic types from all 139 strains at different time points. **(A)** The average concentrations of TcdA determined at 12, 24, 48, and 72 h post inoculation. **(B)** The average concentrations of TcdB determined at 12, 24, 48, and 72 h post inoculation. **(C)** The average concentrations of TcdA and TcdB in all *C. difficile* A+B+CDT+ strains. **(D)** The average concentrations of TcdA and TcdB in all *C. difficile* A+B+CDT-strains.

To confirm toxigenic phenotypes determined by PCR, we further measured toxin production of each strain by ELISA. Corresponding to PCR determination of toxin encoding genes, the toxigenic strains produced either TcdA and/or TcdB, and highest toxin concentration of TcdA and TcdB was detected at 72 hpi (Figure 2; Figures S1, S2 in supplemental materials). Interestingly, it appears that TcdB was produced faster than TcdA (Figures 2C,D). These interesting findings are also in agreement with the previous study (Warny et al., 2005), though the mechanisms behind this phenomenon are not determined yet. In addition, the A+B+CDT+ strains produced more TcdA and TcdB compared to the other strains (Figures 2A,B).

### Antimicrobial susceptibility of the *c. difficile* isolates

The antibiotic susceptibility patterns of the 139 *C. difficile* isolates were summarized in Table 4. As shown in the table, all isolates were susceptible to metronidazole and rifaximin. One isolate (0.72%, *n* = 1) was found to be resistant to vancomycin (MIC = 8 μg/ml), and another isolate (0.72%, *n* = 1) was resistant to fidaxomicin (MIC = 16 μg/ml). For the other antibiotics, 75.54% (*n* = 105), 10.79% (*n* = 15), 5.76% (*n* = 8), 67.63% (*n* = 94), 82.70% (*n* = 115), 45.32% (*n* = 64), 28.06% (*n* = 39), and 28.78% (*n* = 40) of the isolates was resistant to cefoxitin, ceftriaxone, chloramphenicol, ampicillin, clindamycin, erythromycin, gatifloxacin, and moxifloxacin, respectively.

**Table 4:** The antibiotic susceptibility patterns of the 139 *C. difficile* isolates.

**Antibiotics**	**MIC range (μg/ml)**	**No. of isolates with MIC of (**μ**g/ml)**											**Susceptibility profile**			**MIC50 (μg/ml)**	**MIC90 (μg/ml)**
		**≤0.5**	**1**	**2**	**4**	**8**	**16**	**32**	**64**	**128**	**256**	**>256**	**% S**	**% I**	**% R**		
MTZ	≤0.5 to 16	40	24	46	9	19	1						99.28	0.72	0.00	2	8
VAN	≤0.5 to 8	43	51	31	13	1							89.93	9.35	0.72	1	4
RFX	≤0.5 to 4	115	4	12	6	1						1	-	-	0.00	≤0.5	2
FDX	≤0.5 to 16	125	13				1						89.93	-	0.72	≤0.5	1
FOX	≤0.5 to >256	1	1			2	6	24	53	38	4	10	7.190	17.27	75.54	64	256
CRO	≤0.5 to >256	2	1	2	6	13	44	56	8	2	5		48.92	40.29	10.79	32	64
CHL	≤0.5 to 128	2	5	22	57	34	11	6	2				86.33	7.91	5.76	4	16
AMP	≤0.5 to >256	18	27	35	22	11	3	5	3	5	8	2	12.95	19.42	67.63	2	128
CLI	≤0.5 to >256	8	3	3	10	38	18	8	8	4	11	28	10.70	7.19	82.70	16	>256
ERY	≤0.5 to >256	12	25	30	8	1			3		13	47	-	-	45.32	4	128
GAT	≤0.5 to 256	4	7	56	33	1	8	17	11	1	1		48.20	23.74	28.06	4	32
MXF	≤0.5 to 128	3	15	59	22	2	11	16	9	2			55.40	15.83	28.78	2	32

*MTZ, metronidazole; VAN, vancomycin; RFX, rifaximin; FDX, fidaxomicin; FOX, cefoxitin; CRO, ceftriaxone; CHL, chloramphenicol; AMP, ampicillin; CLI, clindamycin; ERY, erythromycin; GAT, gatifloxacin; MXF, moxifloxacine*.

**Figure 3:**
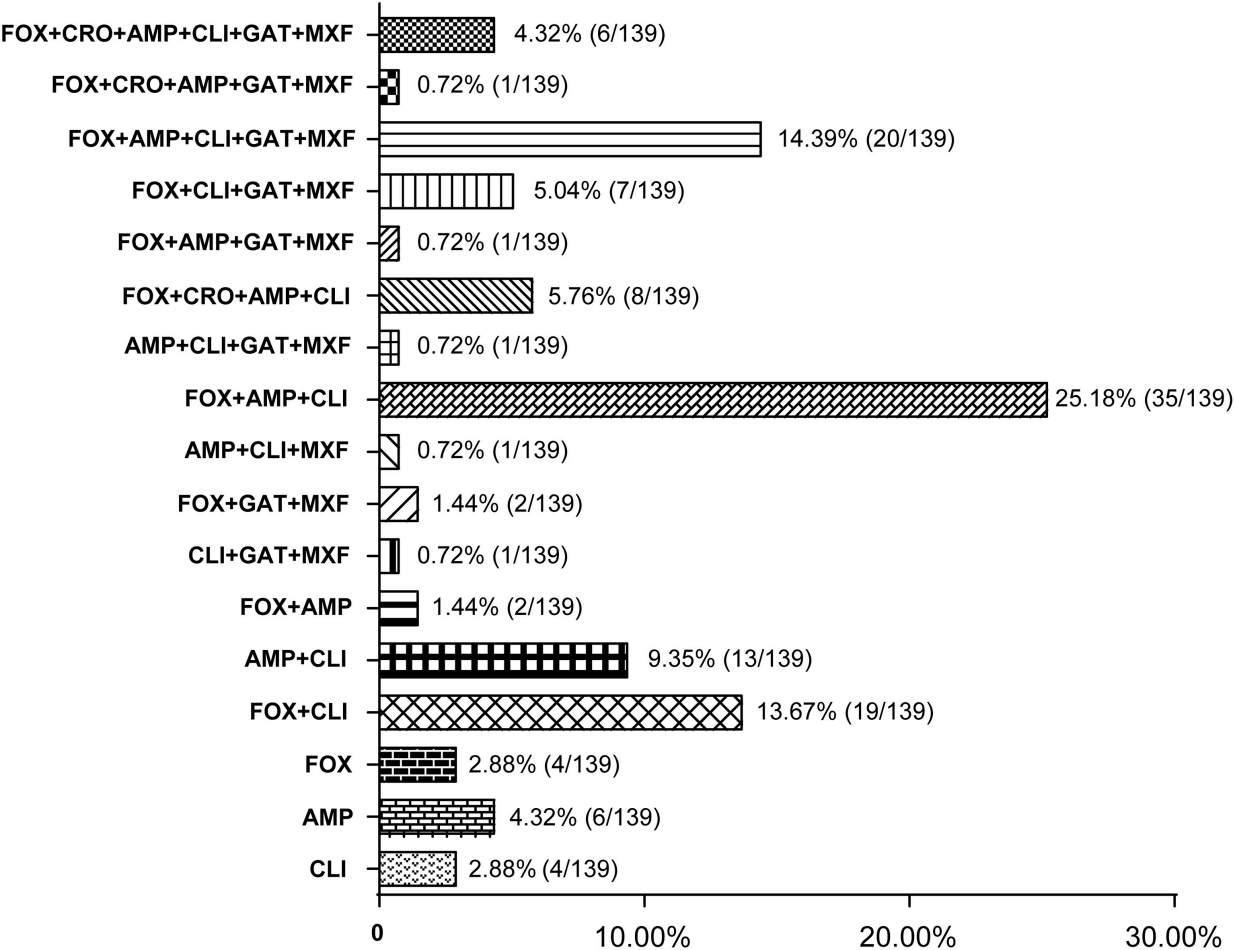
Resistance patterns of *C. difficile* isolates to antibiotics most frequently associated with CDI. The number of isolates that displayed resistance patterns to CDI-associated antibiotics and their percentage were listed at the right side of the column. FOX, cefoxitin; CRO, ceftriaxone; AMP, ampicillin; CLI, clindamycin; GAT, gatifloxacin; MXF, moxifloxacine.

Some antibiotics such as ampicillin, cephalosporins (cefoxitin and ceftriaxone), clindamycin, and fluoroquinolones (gatifloxacin and moxifloxacine) are reported to be most frequently associated with CDI (Leffler and Lamont, 2015). Among the 139 *C. difficile* isolates, 131 strains (92.24%, *n* = 139) were resistant to at least one of those antibiotics, and most of them were resistant to either ampicillin, cefoxitin, or clindamycin. One hundred and seventeen strains (84.17%, *n* = 139) showed resistance to more than two types of antibiotics; and most of them (62.39%, *n* = 73) were resistant to ampicillin and cefoxitin simultaneously (Figure 3). A total of 83 strains (59.71%, *n* = 139) strains had resistance to more than three types of antibiotics; and multiple resistance to ampicillin + cefoxitin + clindamycin was the most common resistance pattern detected among those strains (83.13%, *n* = 69). There were 44 strains (31.65%, *n* = 139) displaying resistance to more than four types of antibiotics, and most of them (63.64%, *n* = 28) were resistant to ampicillin, cefoxitin, gatifloxacin, and moxifloxacin, simultaneously. In addition, 27 strains (19.42%, *n* = 139) displayed resistance to more than five types of antibiotics, and six strains (4.32%, *n* = 139) were resistant to all those six antibiotics, and these six strains included one A+B+CDT+ strain and one A-B-CDT-strain.

All of the A+B+CDT+ isolates were susceptible to metronidazole, vancomycin, rifaximin, and fidaxomicin (Table 5). Percentage of binary toxin-positive strains resistant to cefoxitin, ceftriaxone, chloramphenicol, ampicillin, clindamycin, erythromycin, gatifloxacin, and moxifloxacin was 86.96, 8.69, 8.69, 65.22, 73.91, 73.91, 69.57, and 69.57%, respectively. There were 22 A+B+CDT+ strains (95.65%, *n* = 23) showing resistance to more than 2 types of antibiotics that are very commonly associated with CDI. Among them, 16 strains (69.57%, *n* = 23) displayed resistance to both gatifloxacin and moxifloxacin, simultaneously. Particularly, those strains had a higher MIC50 and MIC90 value of gatifloxacin and moxifloxacine compared to the other isolates (Tables 4, 5).

**Table 5:** The antibiotic susceptibility patterns of the 23 A+B+CDT+ *C. difficile* isolates.

**Antibiotics**	**MIC range (μg/ml)**	**No. of isolates with MIC of (**μ**g/ml)**											**Susceptibility profile**			**MIC50 (μg/ml)**	**MIC90 (μg/ml)**
		**≤0.5**	**1**	**2**	**4**	**8**	**16**	**32**	**64**	**128**	**256**	**>256**	**% S**	**% I**	**% R**		
MTZ	≤0.5 to 4	10	5	6	2								100.00			1	2
VAN	≤0.5 to 2	9	8	2	4								82.61	17.39		1	4
RFX	≤0.5 to 4	15		4	4										0.00	0.5	2
FDX	≤0.5 to 2	17	6										73.91			0.5	1
FOX	2 to >256						1	2	9	8	2	1	4.35	8.69	86.96	64	256
CRO	4 to >256				1		10	10				2	47.83	43.48	8.69	32	32
CHL	1 to 128		1	7	7	5	1	2					86.96	4.35	8.69	4	16
AMP	≤0.5 to >256	2	6	9	1	2		1			1	1	8.69	26.09	65.22	2	32
CLI	≤0.5 to >256	1		1	4	5		1	1	2	3	5	8.70	17.39	73.91	32	>256
ERY	0.5 to >256	1	2	1	2						4	13			73.91	>256	>256
GAT	≤0.5 to 256		1	1	5		2	8	5		1		8.69	21.74	69.57	32	64
MXF	≤0.5 to 128		1	3	3		2	9	4	1			17.39	13.04	69.57	32	64

*MTZ, metronidazole; VAN, vancomycin; RFX, rifaximin; FDX, fidaxomicin; FOX, cefoxitin; CRO, ceftriaxone; CHL, chloramphenicol; AMP, ampicillin; CLI, clindamycin; ERY, erythromycin; GAT, gatifloxacin; MXF, moxifloxacine*.

## Discussion

CDI is a toxin-mediated disease, and the expression of two large clostridial toxins A (TcdA) and B (TcdB) is considered causes of CDI symptoms (Voth and Ballard, 2005; Elliott et al., 2017). While three main toxigenic types (A+B+, A+B-, A-B+) are defined based on the possession of toxin encoding genes *tcdA* and *tcdB*, they have different detection rates in clinical incidence of CDI with A+B+ being the most common toxigenic types (Jalali et al., 2012; Snydman et al., 2015; Cheng et al., 2016; Singh et al., 2017). Consistent with those studies, ~89.21% of the TGH clinical isolates investigated in this study were A+B+ strains, while only 2.88% of them were A-B+ strains, suggesting that A+B+ is still the predominant toxigenic type in clinic. However, we did not detect A+B-strains in this investigation. It appears that, this toxigenic type (A+B−) is also rarely seen in other epidemical studies (Jalali et al., 2012; Cheng et al., 2016). A toxinotyping and sequencing investigation of *C. difficile* isolates from patients in a Tertiary Care Hospital of Northern India identified 13 strains (10.7%) only carrying *tcdA* (Singh et al., 2017). Those data suggest that toxin B is more associated with the development of CDI in clinic. This speculation could be also supported by an *in vivo* study in hamster models that provides evidence that toxin B, not toxin A, is essential for virulence (Lyras et al., 2009).

Besides Tcd A and Tcd B, ~20% of *C. difficile* strains are found expressing the binary toxin (CDT) (Eckert et al., 2015). Correspondingly, the percentage of *C. difficile* isolates described in this study that possess the CDT encoding genes was 18.71% (26/139). This toxigenic type pattern (A+B+CDT+) also has a relatively low detection rate in clinic, and is commonly seen in some specific ribotypes of *C. difficile* such as the 027 and 078 strains (Álvarez-Pérez et al., 2017; Aschbacher et al., 2017; Beran et al., 2017). It has been reported that the CDT-positive strains of *C. difficile* cause higher fatality rates than those CDT-deficient strains, and the prevalence of the 027 strains that produce binary toxin is widely accepted to have association in part with the significant increase in morbidity and mortality related to CDI (Bacci, 2011; Napolitano and Edmiston, 2017). We also found that A+B+CDT+ strains produced more TcdA and TcdB compared to the other strains (Figures 2A,B). A previous study found that the *cdtR* gene harbored in CDT encoding locus (CdtLoc) positively regulated the production of toxins A and B in 027 strains (Lyon et al., 2016). The higher concentrations of TcdA and TcdB produced by the A+B+CDT+ strains might be associated with the CdtLoc harbored by them. We also detected three CDT positive strains that possess *tcdB* but lack *tcdA* (A-B+CDT+); however, this toxigenic type pattern is rarely seen in clinic. Further analyses are required for the determination of their pathogenesis.

Antibiotic use is proposed to be the most important risk for CDI (Leffler and Lamont, 2015; Napolitano and Edmiston, 2017; Peng et al., 2017). Disruption of the intestinal microbiota, typically but not only caused by antibiotics, is essential for the establishment of *C. difficile* and toxin production (Elliott et al., 2017). Epidemical data showed that resistance to clindamycin (8.3 to 100%), cephalosporins (51%), erythromycin (13 to 100%), and fluoroquinolones (47%) is commonly seen in *C. difficile* clinical isolates within the past 15 years (2000–2015) (Spigaglia, 2016). Resistance to those antibiotics was also common in the isolates investigated in this study. Our data revealed that 82.70 and 45.32% of the strains were resistant to clindamycin and erythromycin, respectively. In addition, 75.54% of the strains showed resistance to the second-generation cephalosporins (cefoxitin) while 10.97% of the strains were resistant to the third-generation cephalosporins (ceftriaxone). Moreover, 28.06 and 28.78% of the strains displayed resistance to the fourth-generation fluoroquinolones gatifloxacin, and moxifloxacin, respectively (Table 4). Those data suggest that antibiotic resistance of *C. difficile* remains prevailing. More worrisome, most of the *C. difficile* isolates investigated in this study showed resistance to multiple antibiotics, with AMP+FOX, AMP + FOX + CLI, AMP + FOX + GAT + MXF being the most common multiple resistance patterns (Figure 2). All ampicillin, clindamycin, cephalosporins, and fluoroquinolones are known to promote CDI (Leffler and Lamont, 2015; Peng et al., 2017). A high percentage of *C. difficile* isolates resistant to those antibiotics increases the risk of CDI.

Resistance profiles of the isolates to metronidazole, vancomycin, rifaximin, and fidaxomicin should also receive more attention. Both metronidazole and vancomycin are recommended therapies of choice for CDI (Leffler and Lamont, 2015). Although no isolates were found to have full resistance to metronidazole, there were still one strain intermediately resistance to metronidazole and 19 strains having MIC of 8 μg/ml (Table 4). Vancomycin is a first-line option in severe CDI (Gerding et al., 2016). While the majority of *C. difficile* isolates were still susceptible to vancomycin, one resistant strain (0.72%) was detected, and the MIC of vancomycin to this isolate was 8 μg/ml. In fact, resistance of *C. difficile* to vancomycin has been reported during the past years (Goudarzi et al., 2013; Adler et al., 2015; Freeman et al., 2015; Snydman et al., 2015). Even though vancomycin resistance level is unlikely to affect primary treatment efficacy for CDI (Baines and Wilcox, 2015), these data still suggest a potentially serious problem for vancomycin therapy of CDI in the future. Both rifaximin and fidaxomicin are proposed as effective alternatives for CDI (Leffler and Lamont, 2015), and fidaxomicin has been approved by the US Food and Drug Administration for its use in CDI treatment following oral vancomycin (Lancaster and Matthews, 2012). Correspondingly, no isolate investigated in this study was found resistance to rifaximin and only one isolate was resistant to fidaxomicin (Table 4). Those findings, in turn, support the potential use of rifaximin and fidaxomicin in treating CDI.

In conclusion, we tested antibiotic resistance and toxin production of *C. difficile* isolates from patients diagnosed with CDI in 2016. Even though A+B+CDT-is still the predominant toxigenic type in clinic, some other toxigenic types such as A+B+CDT+, A-B+CDT+, and A-B+CDT-are also defined. Among the two toxins expressed by *C. difficile*, TcdB is produced faster than TcdA, and CDT might have a positive role in regulating the production of toxins A and B. Our findings also show that antibiotic resistance remains a serious problem for *C. difficile*, which is of concern. Determination of sequences, ribotypes, sporulation, germination, biofilm production, and many others will be our next phase of continued studies for the selected multiple antibiotic-resistant *C. difficile* strains and unique toxin-type strains. In the next step, we also intend to do a follow up study to correlate the severity of CDI with toxin production profiles as well as antibiotic resistance patterns.

## Author contributions

ZP, SA, and XS participated in the conception and design of the work. AA and SA contributed to the bacterial isolation and collection. ZP performed the experiments. XS supervised the laboratory work with the bacterial isolates and the MIC tests. ZP, SA, AA, and XS participated in the manuscript writing. All authors read and approved the final manuscript.

### Conflict of interest statement

The authors declare that the research was conducted in the absence of any commercial or financial relationships that could be construed as a potential conflict of interest.

## Supplementary material

Figure S1 The concentration of TcdA produced by each of the TGH strains at 12, 24, 48, and 72 h post inoculation.

Figure S2 The concentration of TcdB produced by each of the TGH strains at 12, 24, 48, and 72 h post inoculation.
