# Supplementary figures and images for: Antibiotic Resistance and Toxin Production of Clostridium difficile Isolates from the Hospitalized Patients in a Large Hospital in Florida
Source: Front Microbiol. 2017 Dec 22;8:2584. doi: 10.3389/fmicb.2017.02584 (PMC5744170; doi:10.3389/fmicb.2017.02584)

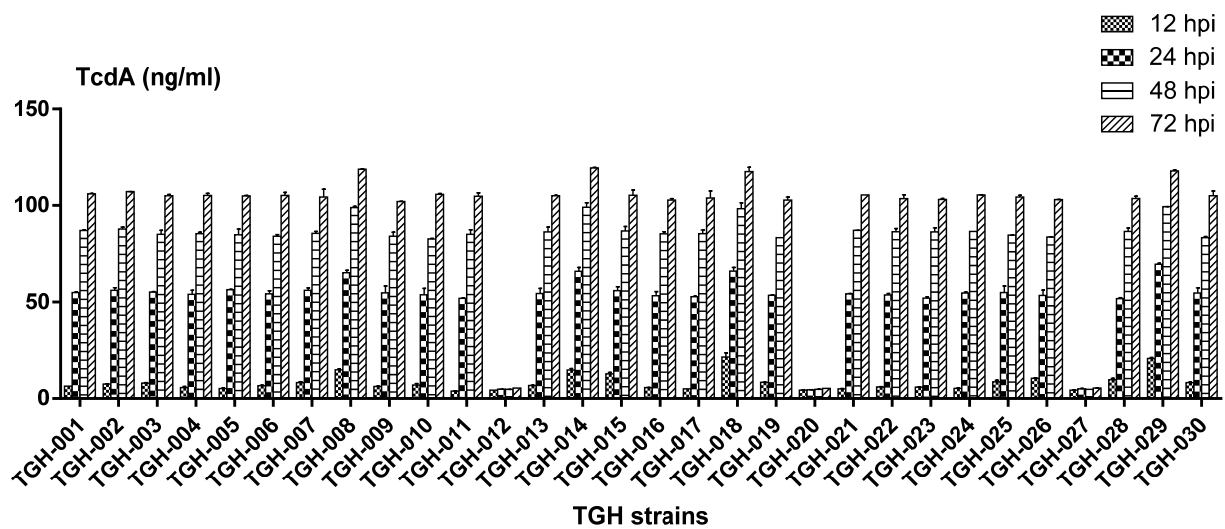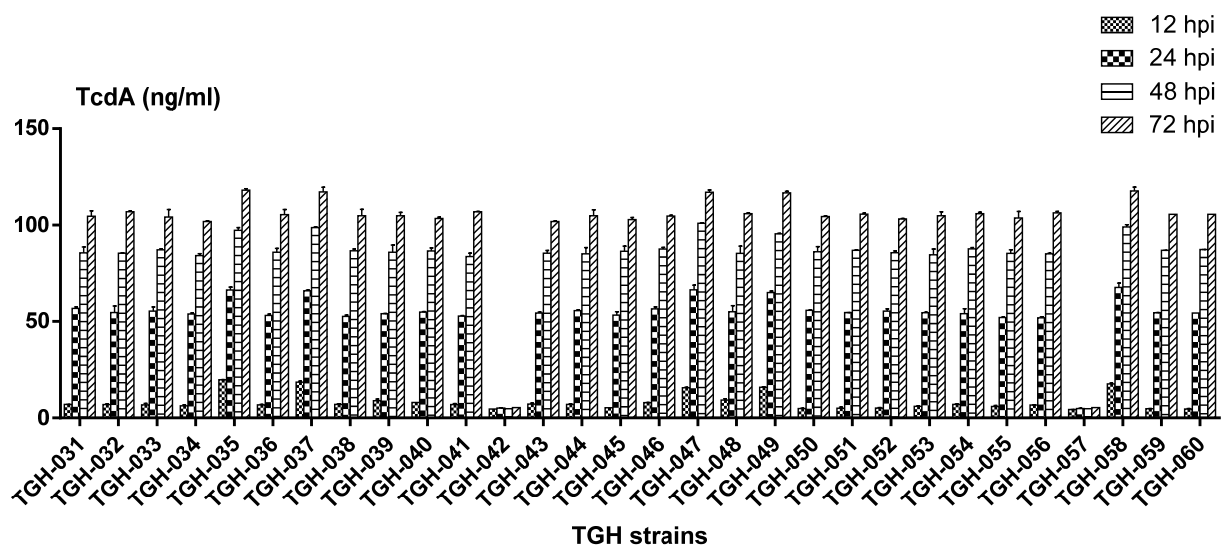

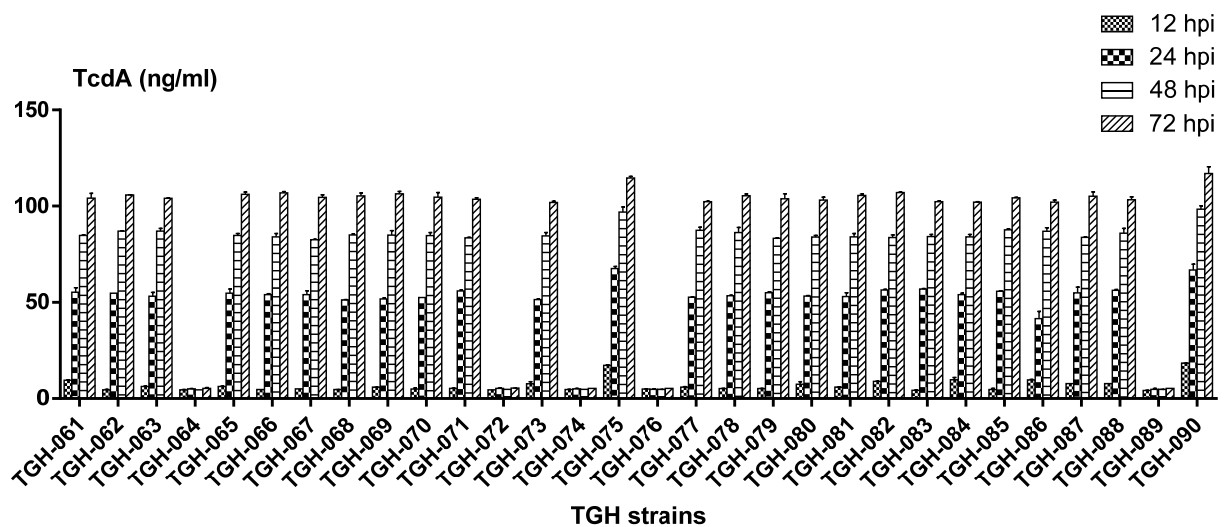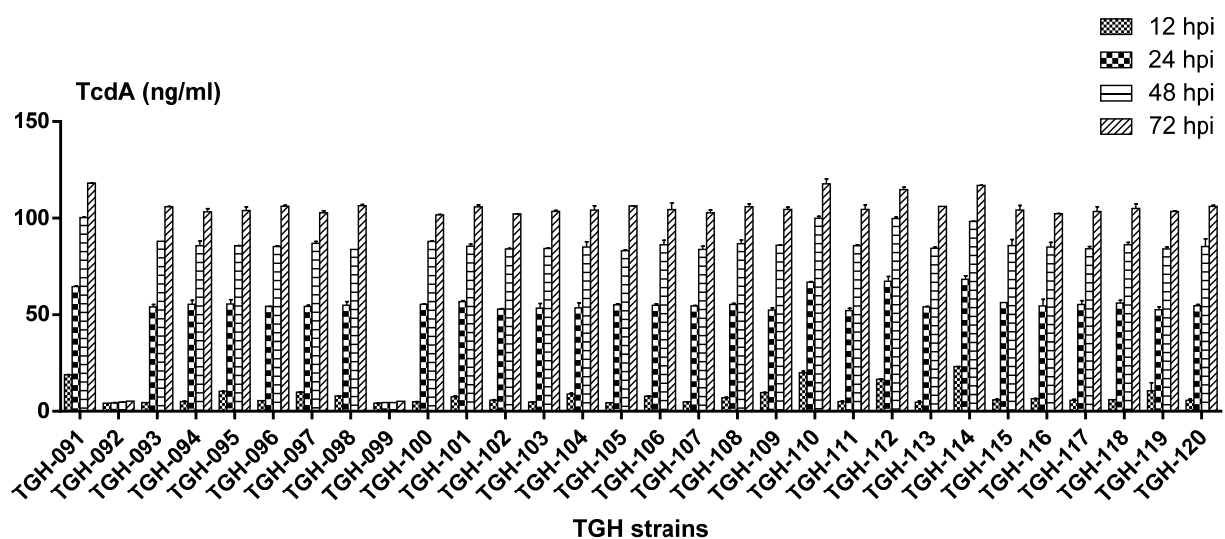

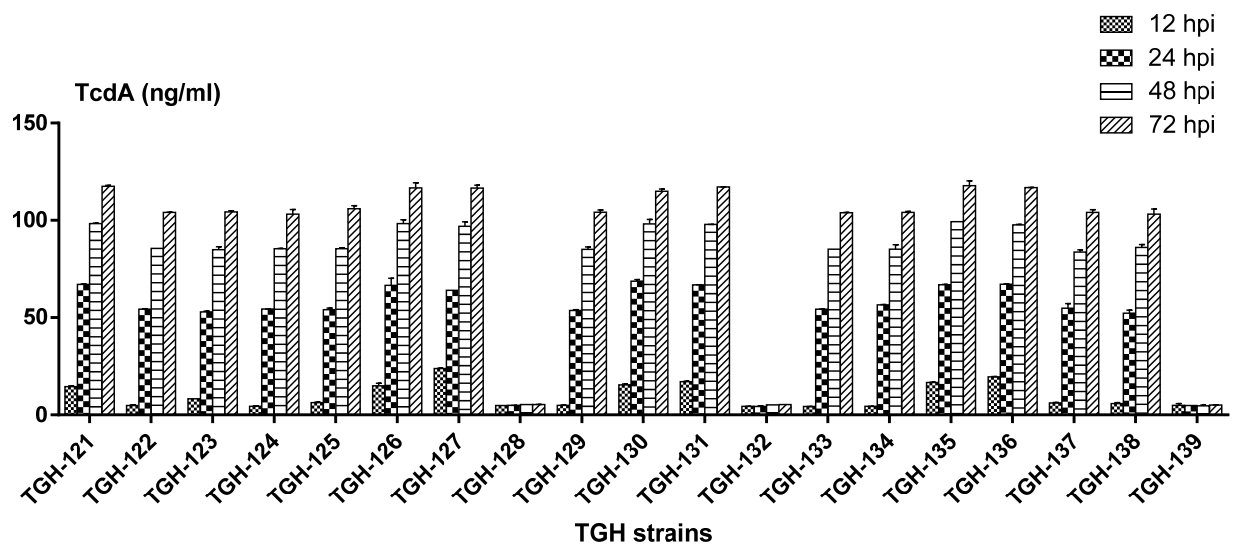

Supplement: Figure S1 — The concentration of TcdA produced by each of the TGH strains at 12, 24, 48, and 72 h post inoculation. [file Image1.PDF]

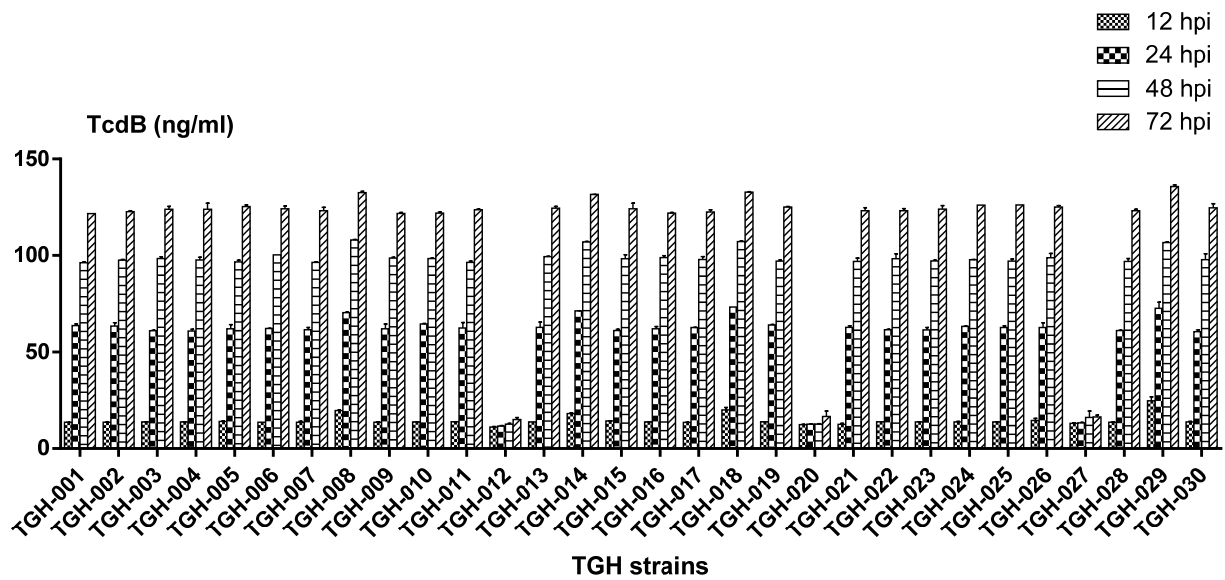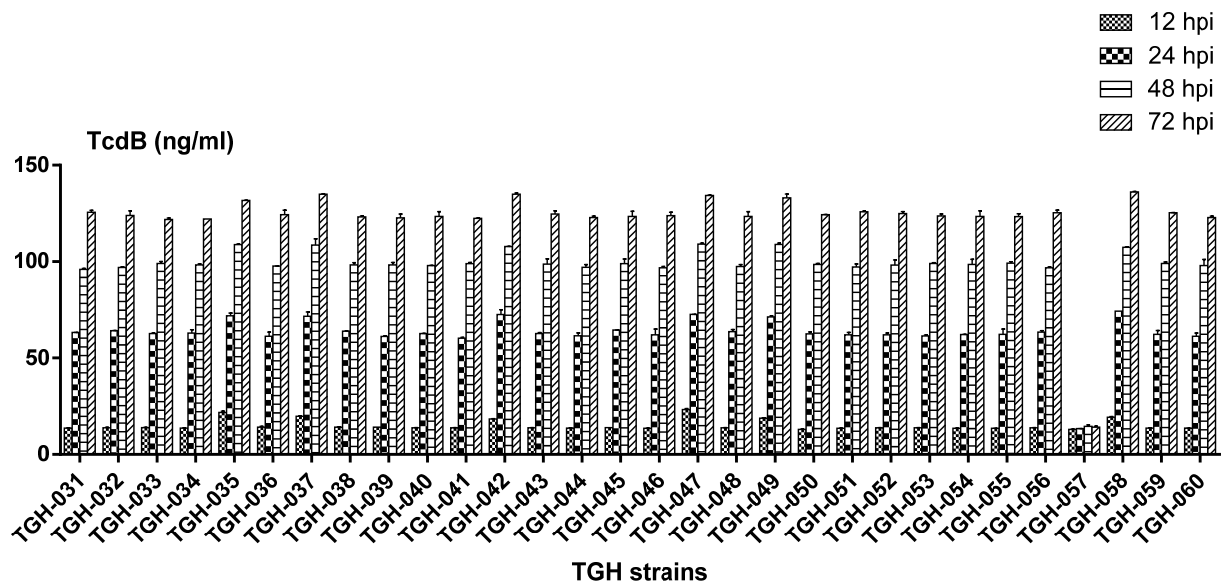

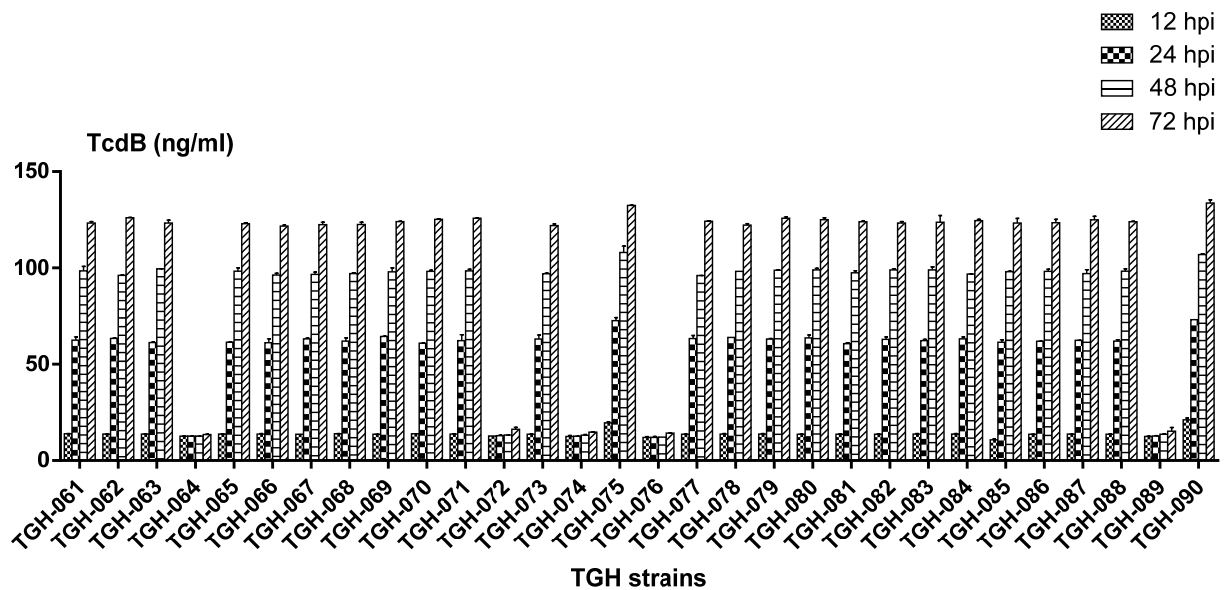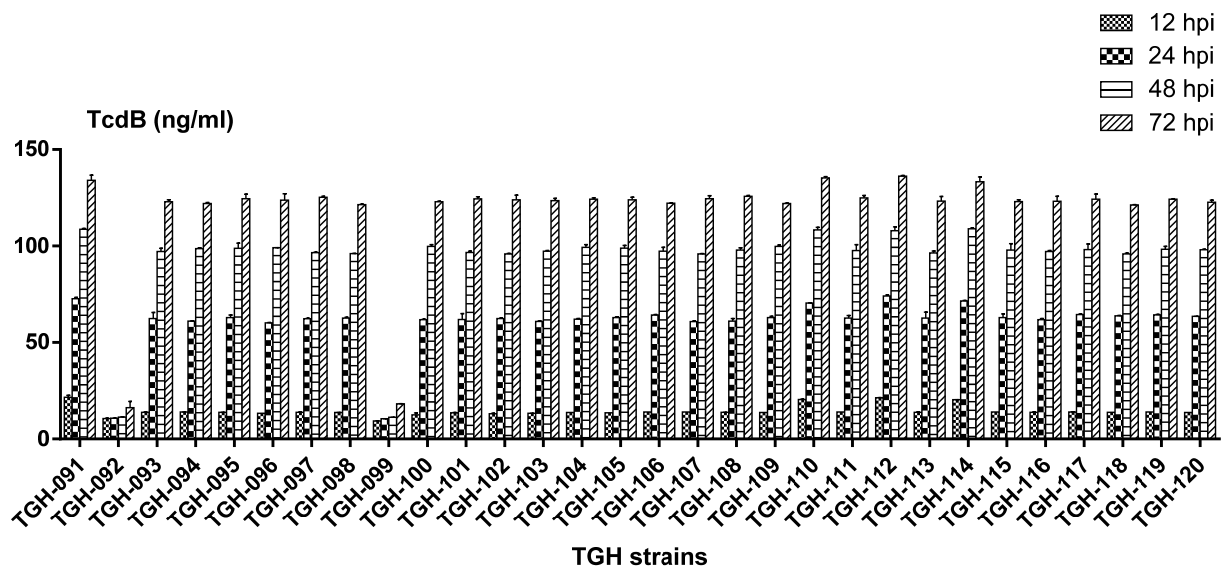

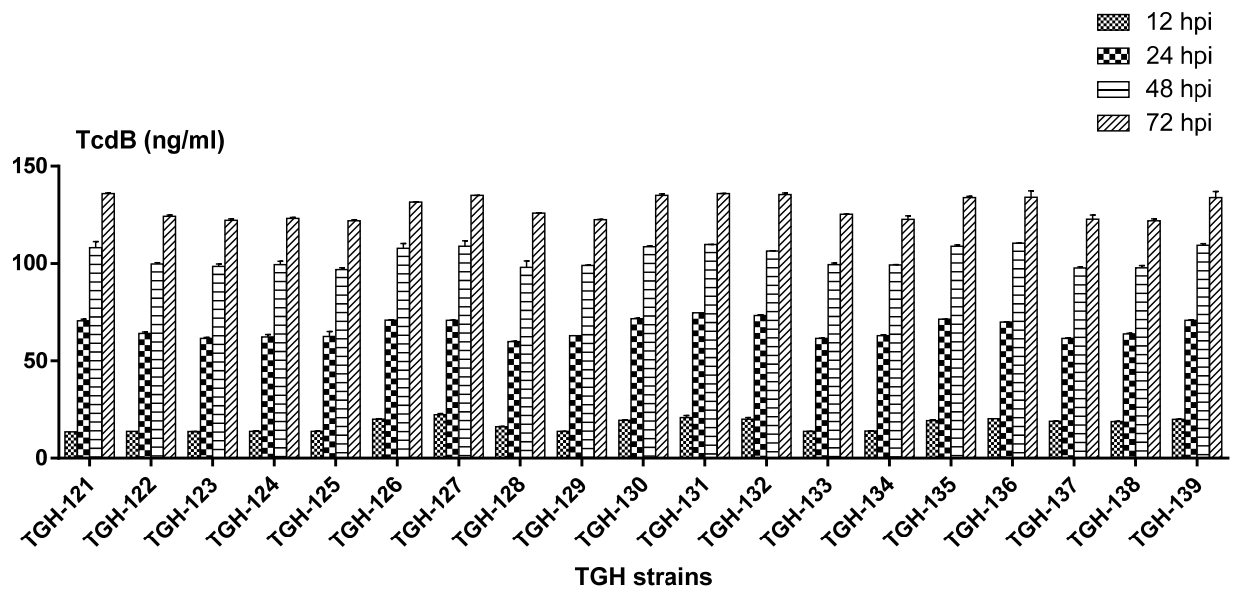

Supplement: Figure S2 — The concentration of TcdB produced by each of the TGH strains at 12, 24, 48, and 72 h post inoculation. [file Image2.PDF]
